# Supplementary material for: Antimicrobial-producing bacteria from fish epidermal mucus alter the fish epidermal bacterial flora and host resistance to infection
Source: Appl Environ Microbiol. 2025 Oct 30;91(11):e01450-25. doi: 10.1128/aem.01450-25 (PMC12628852; doi:10.1128/aem.01450-25)
Supplement: Supplemental material — Figures S1 to S10 and Tables S1 and S2. [file aem.01450-25-s0004.pdf]

Supplemental materials

Antimicrobial-producing Bacteria from Fish Epidermal Mucus Alter the Fish Epidermal Bacterial Flora and Host Resistance to Infection

Hajime Nakatani[1] and Katsutoshi Hori[1]*

[1]Graduate School of Engineering, Nagoya University
Furo-cho, Chikusa, Nagoya, Aich 464-8603 Japan

*Corresponding author: Katsutoshi Hori (khori@chembio.nagoya-u.ac.jp)

Running title: Antimicrobial Bacteria Isolated from Fish Epidermal Mucus

Contents

Fig. S1: Identification of bacterial species relating to strain C6 and KH-ZF1
Fig. S2: *Pseudomonas mosselii* KH-ZF1 strain inhibits the growth of various fish pathogenic bacteria
Fig. S3: Colony formation of KH-ZF1::mCherry collected from zebrafish epidermal mucus after administration.
Fig. S4: Change in the epidermal mucus bacterial flora on the fish without strain KH-ZF1 administration.
Fig. S5: Influences of the strain KH-ZF1 administration on the bacterial flora in gills and intestinal content.
Fig. S6: Schedule for the strain KH-ZF1 administration in the infection prevention experiments.
Fig. S7: MS of an antimicrobial substance from strain KH-ZF1.
Fig. S8: NMR spectra of an antimicrobial substance from strain KH-ZF1.
Fig. S9: Schedule for Fluviol C administration experiment.
Fig. S10: Confirmation of the appropriate concentration of Fluviol C for administration to zebrafish.

Table S1: X-ray crystallographic data for an antimicrobial substance from strain KH-ZF1
Table S2: Bacterial strains used in this study.

References for Table S2

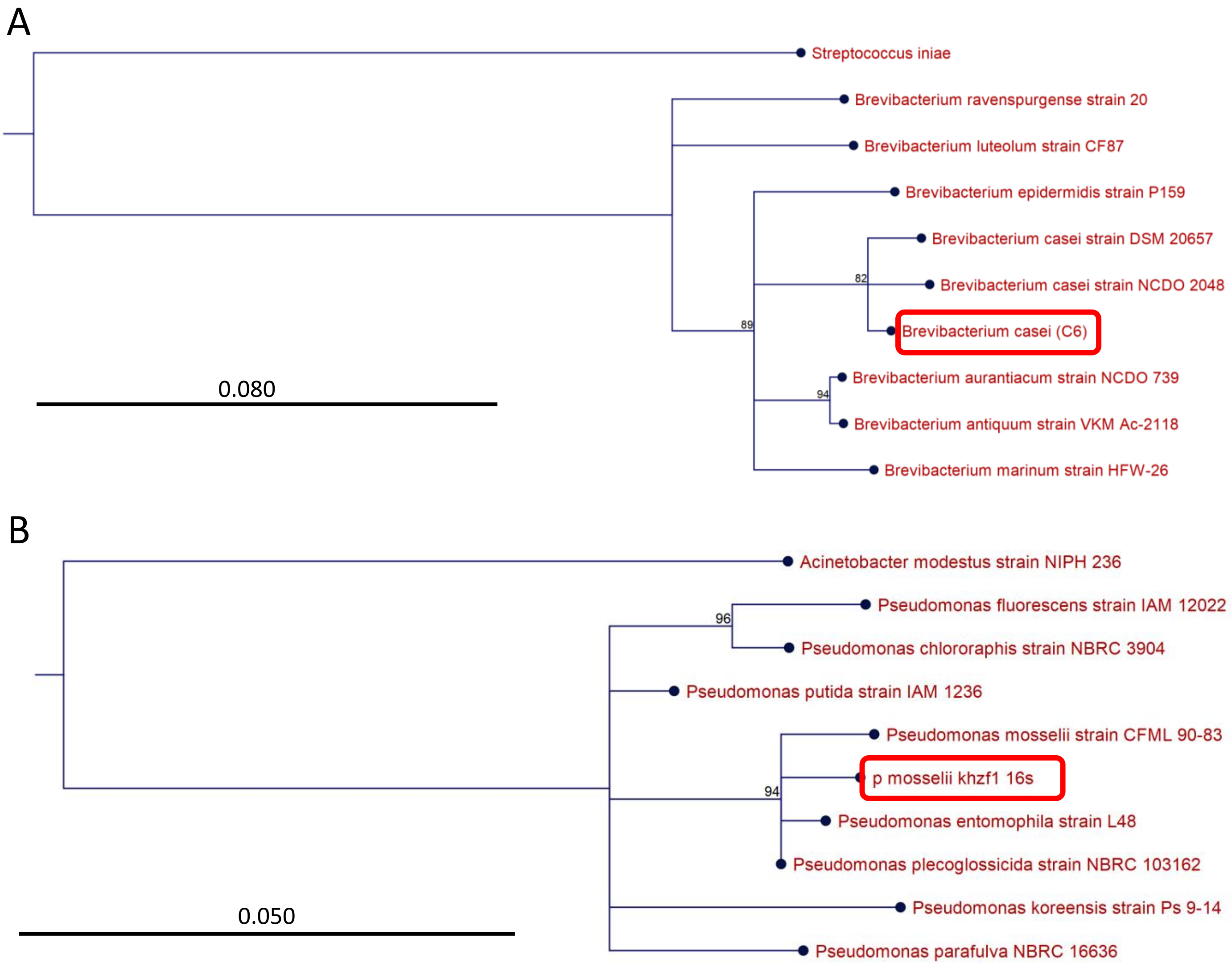

Fig. S1. Identification of bacterial species relating to strain C6 and KH-ZF1. The sequence of 16S rRNA gene (C6 and KH-ZF1) was compared with related bacterial species, and drew phylogenetic trees from sequence similarity. A) C6 : Closely related to *Brevibacterium casei* strain NCDO 2048 (identity 98.83%). B) KH-ZF1: Closely related to *Pseudomonas mosselii* CFML 90-83 (identity 99.74%).

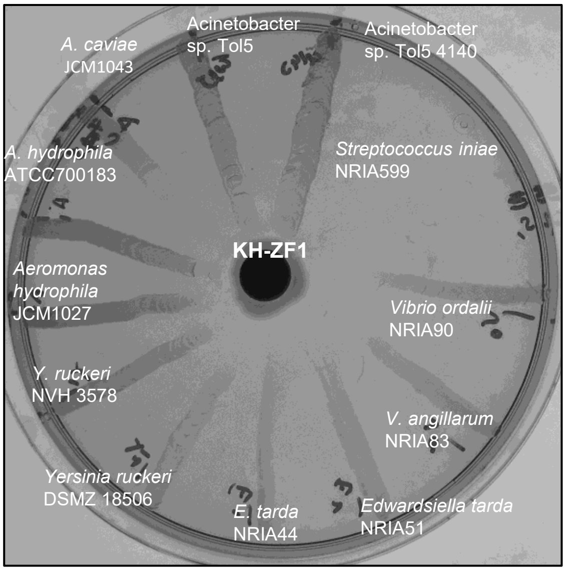

Fig. S2. *Pseudomonas mosselii* KH-ZF1 strain inhibits the growth of various fish pathogenic bacteria. Growth inhibition of multiple fish pathogens by the *Pseudomonas mosselii* KH-ZF1 strain was evaluated. Acinetobacter sp. Tol 5, a non-pathogenic strain, was included as a negative control.

Fig. S3

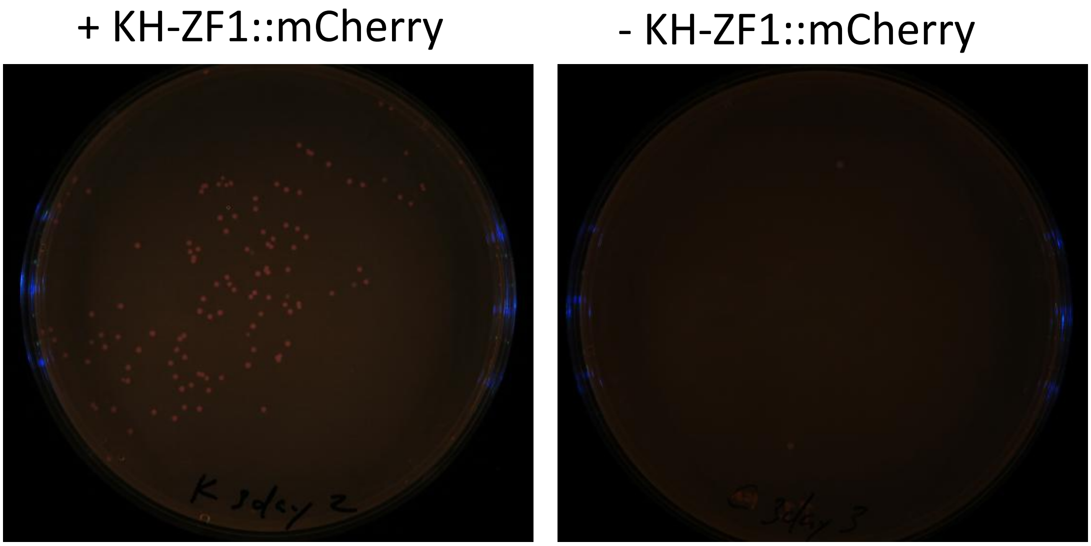

Fig. S3. Colony formation of KH-ZF1::mCherry collected from zebrafish epidermal mucus after administration. The epidermal mucus was collected from zebrafish administered KH-ZF1::mChery (+ KH-ZF1::mCherry) or control fish (- KH-ZF1::mCherry) for CFU count. The mucus was collected at 3 days after second dose in administration experiment shown in fig.3 (day 4 in fig 3), and culture on selection medium containing kanamycin. The colony producing mCherry were observed under blue LED light.

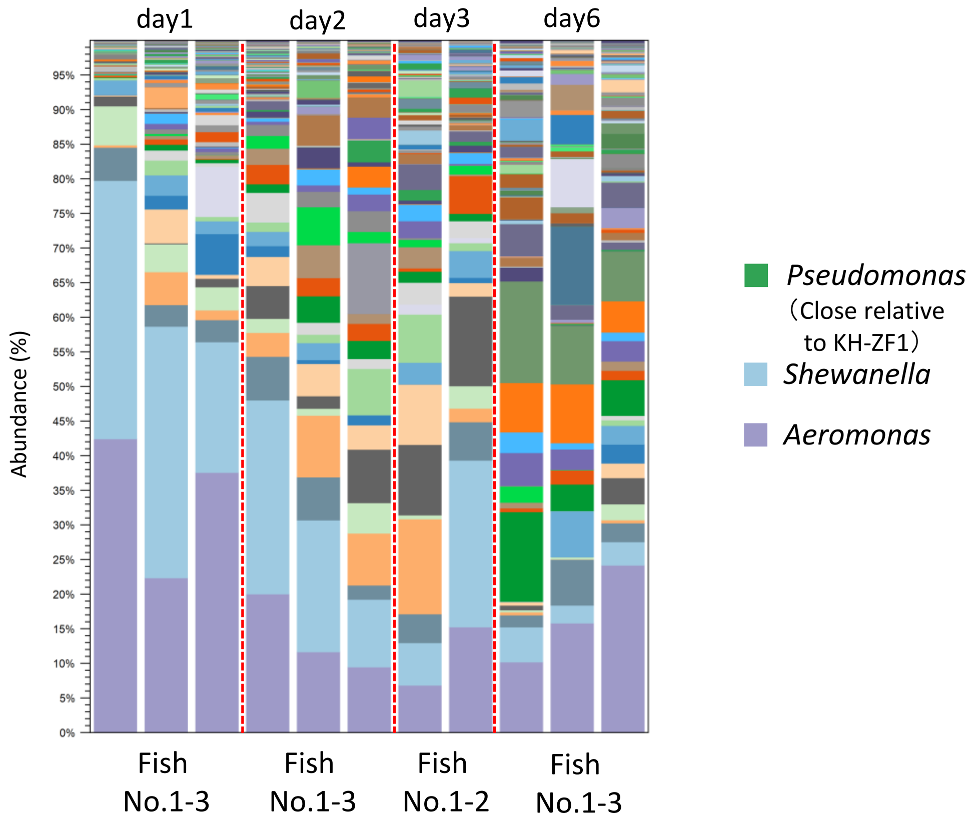

Fig. S4. Change in the epidermal mucus bacterial flora on the fish without strain KH-ZF1 administration. The bacterial floras of the epidermal mucus were chronologically analyzed from the day1 of infection prevention experiment to day 6 by OTU clustering.

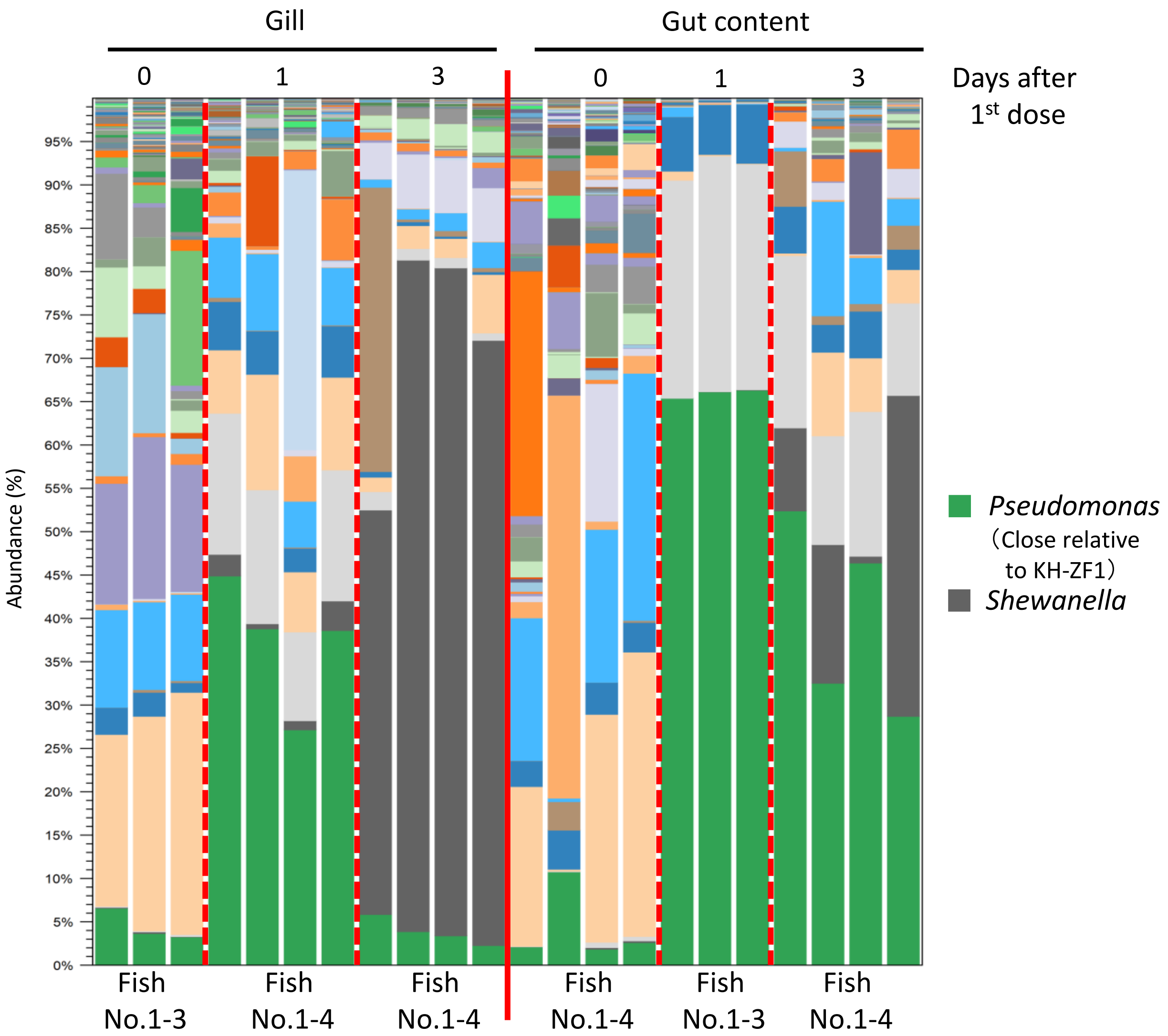

Fig. S5 Influences of the KH-ZF1 administration on the bacterial flora in gills and intestinal content. The bacterial floras of gills and gut content were chronologically analyzed after twice administration of KH-ZF1 at day 0 and day 1

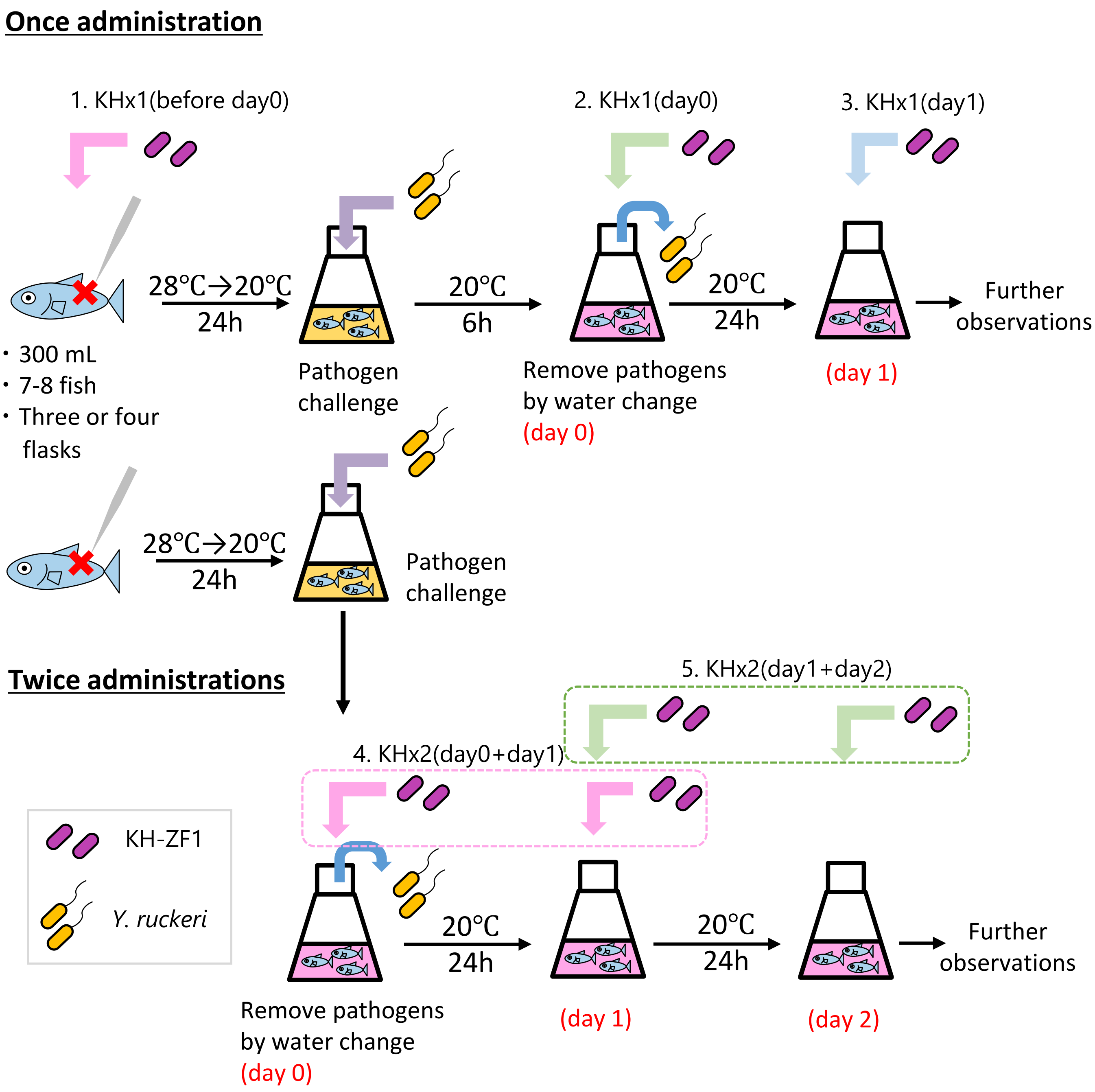

Fig. S6. Schedule for strain KH-ZF1 administration in the infection prevention experiments.
Strain KH-ZF1 was administrated once or twice before or after pathogen challenge. Five administration conditions were tested. Final OD_{600} of strain KH-ZF1 in rearing water after administration was adjust to 0.01.

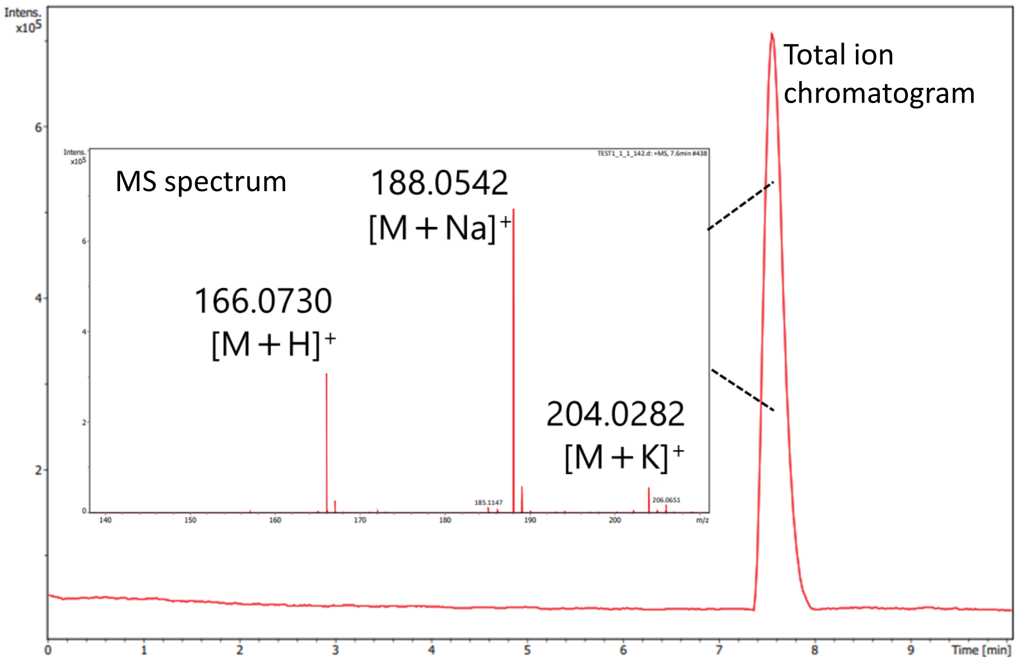

Fig. S7. MS of an antimicrobial substance from strain KH-ZF1. Total ion chromatogram and MS of the fraction containing an antimicrobial substance. Three major MS peaks (m/z =166.0730, 188.0542, and 204.0282) obtained from ions around 7.6 minutes were shown.

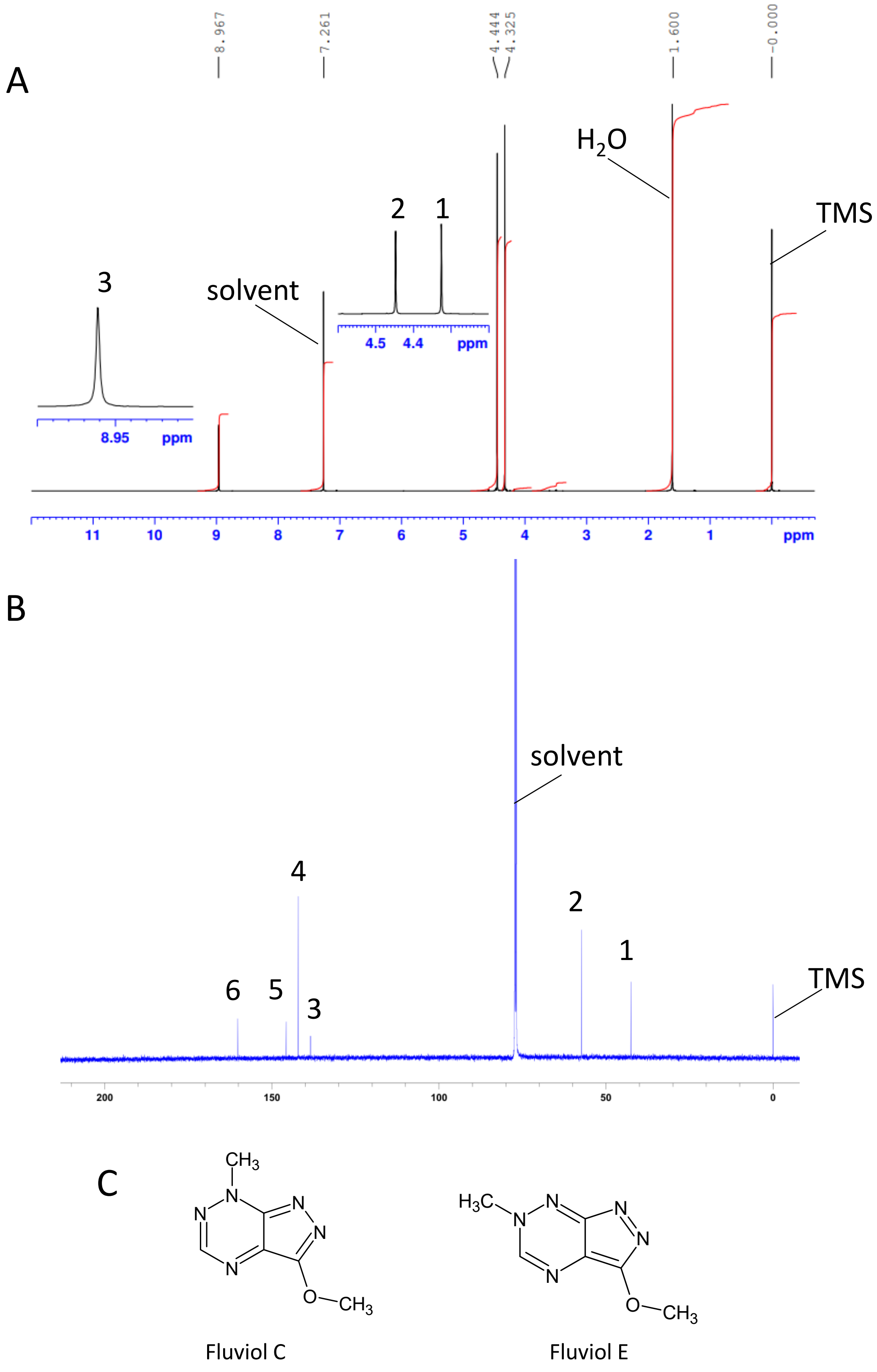

Fig. S8. NMR spectra of an antimicrobial substance from strain KH-ZF1. A) ^{1}H NMR spectra of NMR spectra of antimicrobial substance from strain KH-ZF1. No.1 and 2, singlet peaks at 4.33 and 4.44 ppm from protons of methyl group. No.3, singlet peak at 8.97 ppm from aromatic proton. B) ^{13}C NMR spectra of antimicrobial substance from strain KH-ZF1. No.1 and 2, peaks from methyl carbons. No.3-No.6, peaks from aromatic carbons. C) Possible chemical structures of natural compounds with composition formula, $C_6H_7N_5O$.

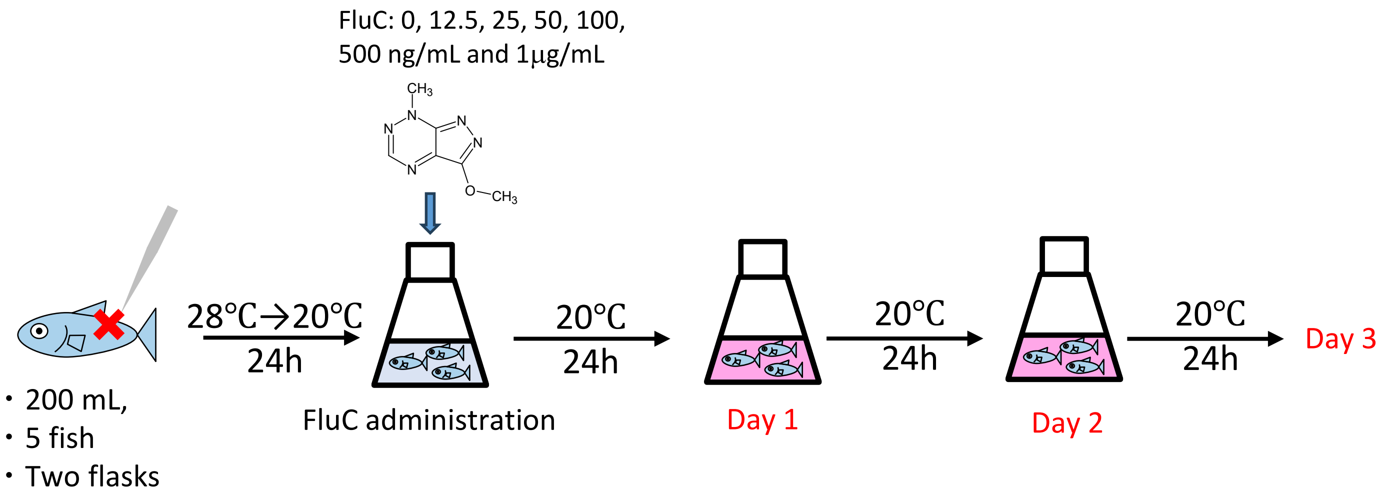

Fig. S9. Schedule for FluC administration experiment.
FluC was administrated after injury and temperature change at several concentration, and the number of survive fish was recorded for three days.

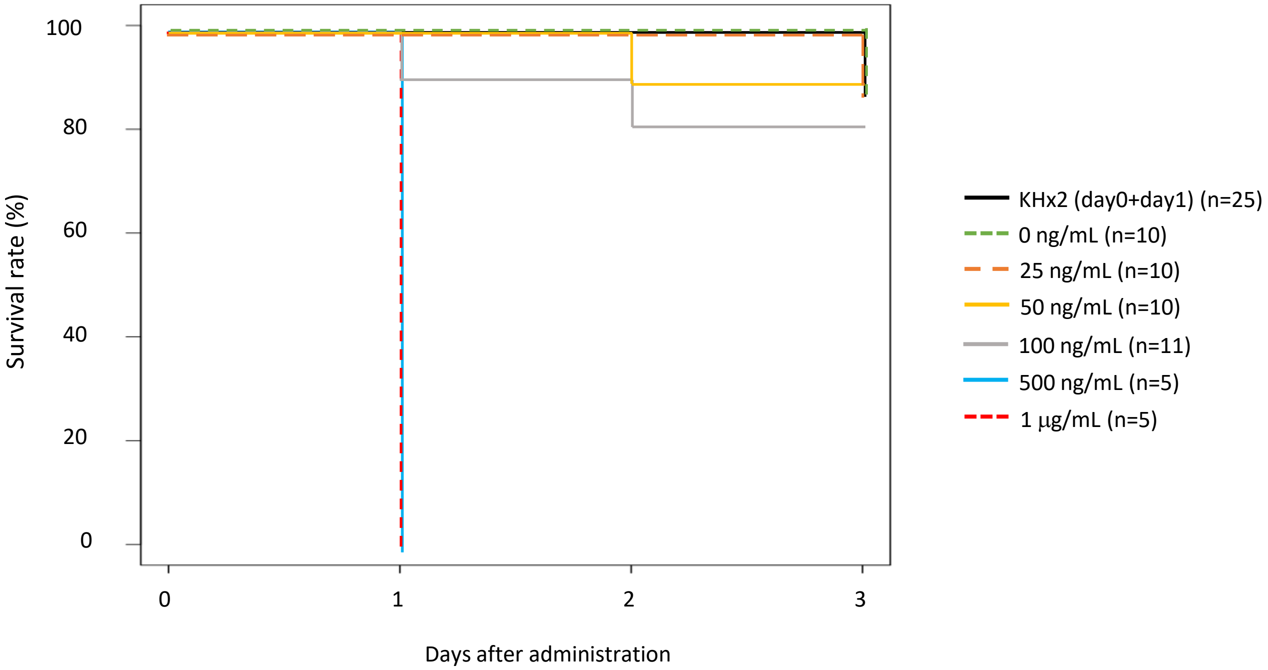

Fig. S10. Confirmation of the appropriate concentration of Fluviol C for administration to zebrafish. The number of surviving fish after administration of Fluviol C or strain KH-ZF１strain was observed for 3 days. KHx2: Double-dose administration of KH-ZF1 cells at day0 and day1.

Table S1. X-ray crystallographic data for an antimicrobial substance from strain KH-ZF1

Empirical formula	$C_6H_7N_5O$
Formula Weight	165
Crystal Color, Habit	gray, chip
Crystal Dimensions	0.400 X 0.100 X 0.100 mm
Crystal System	monoclinic
Lattice Type	C-centered
Lattice Parameters	a = 9.135(3) Å
	b = 15.277(5) Å
	c = 6.456(2) Å
	β = 127.048(5) °
	V = 719.1(4) $Å^3$
Space Group	Cc (#9)
Z value	4
Density (calculated)	1.525 g/cm^3
F_{000}	344
μ(MoKα)	1.135 cm^{-1}
Radiation	MoKα (λ = 0.71075 Å)
	multi-layer mirror monochromated
Temperature	123.0 K
ω oscillation Range (χ=45.0, ϕ=0.0)	-105.0 ~75.0°
$2\theta_{max}$	55.0°
No. of Reflections Measured	Total: 3049
	Unique: 1347 (R_{int} = 0.0720)
	Parsons quotients (Flack x parameter):
Corrections	Lorentz-polarization
	Absorption
	(trans. factors: 0.835 - 0.989)
Structure Solution	Direct Methods (SHELXT Version 2014/5)
Refinement	Full-matrix least-squares on F2
$2\theta_{max}$ cutoff	55.0°
Anomalous Dispersion	All non-hydrogen atoms
No. Observations (All reflections)	1347
No. Variables	109
Reflection/Parameter Ratio	12.36
Residuals: R1 (I>2.00s(I))	0.0494
Residuals: R (All reflections)	0.0512
Residuals: wR2 (All reflections)	0.1233
Goodness of Fit Indicator	1.057
Flack parameter (Parsons' quotients = 474)	0.8(10)
Max Shift/Error in Final Cycle	0.000
Maximum peak in Final Diff. Map	0.34 e /$Å^3$
Minimum peak in Final Diff. Map	-0.33 e /$Å^3$

Table. S2 Bacterial strain used in this study (except for *Y. ruckeri* and KH-ZF1).

Bacterial strains	Culture medium	Experiments	References
Acinetobacter baumanii ATCC19606	NB2, MHB[1)]	MIC	Purchase from ATCC[2)]
Acinetobacter sp. Tol 5	LB, NB2	Cross streak	(1)
Acinetobacter sp. Tol 5 4140	LB, NB2	Cross streak	(2)
Aeromonas caviae JCM1043	NB2	Cross streak	Purchase from JCM[3)]
A. hydrophila ATCC 700183	NB2, MHB	Cross streak<br>MIC	Purchase from ATCC
A. hydrophila JCM1027	NB2	Cross streak	Purchased from JCM
A. hydrophila NRIA14	NB2	Cross streak	Provided from FRA[4)]
Bacillus subtilis isw1214	NB2	MIC	Purchase from TaKaRa
Edwardsiella tarda NRIA51	NB2	Cross streak	Provided from FRA
E. tarda NRIA44	NB2	Cross streak<br>MIC	Provided from FRA
Escherichia coli NBRC106373	LB, MHB	MIC	Provided from NBRC[5)]
E. coli S17-1 λpir::pBSL118_23119-mCherry	LB+Amp*+Km**	Conjugal transfer to KH-ZF1	This study
Flavobacterium columnare JCM 21327	Enriched cytophaga Modified Shieh Agar	Cross streak	Purchase from JCM
Pseudomonas aeruginosa PAO1	NB2, MHB	MIC	-
P. putida T57	NB2	Co-culture	-
Streptococcus iniae NRIA599	TSB	Cross streak<br>MIC	Provided from FRA
Vibrio angillarum NRIA83	Zobel2216E, NB2	Cross streak<br>MIC	Provided from FRA
V. ordalii NRIA90	Zobel2216E, NB2	Cross streak<br>MIC	Provided from FRA
Candida cylindracea SL1B2	YPAD[6)]	MIC	(3)
Saccaromyces cerevisiae Y2HGOLD	YPAD	MIC	Purchase from Clontech

1) Mueller Hinton broth 2) American type culture collection 3) Japan collection of microorganisms 4) Japan fisheries research and education agency 5) Biological Resource Center,NITE 6) Yeast Extract Peptone Adenine Dextrose Broth *Ampicillin, **Kanamycin

References for Table S2

(1) Hori K, Yamashita S, Ishii S, Kitagawa M, Tanji Y, Unno H: Isolation, characterization and application to off-gas treatment of toluene-degrading bacteria. *J Chem Eng Japan.* 2001, 39: 175-184.

(2) Ishikawa, M., Hori, K. A new simple method for introducing an unmarked mutation into a large gene of non-competent Gram-negative bacteria by FLP/FRT recombination. *BMC Microbiol* 13, 86 (2013). https://doi.org/10.1186/1471-2180-13-86

(3) Matsuoka, H., Miura, A. & Hori, K. Symbiotic effects of a lipase-secreting bacterium, *Burkholderia arboris* SL1B1, and a glycerol-assimilating yeast, *Candida cylindracea* SL1B2, on triacylglycerol degradation. *J. Biosci. Bioeng*. 107, 401–408 (2009).
