# Supplementary material for: Antimicrobial-producing bacteria from fish epidermal mucus alter the fish epidermal bacterial flora and host resistance to infection
Source: Appl Environ Microbiol. 2025 Oct 30;91(11):e01450-25. doi: 10.1128/aem.01450-25 (PMC12628852; doi:10.1128/aem.01450-25)
Supplement: Supplemental legends — Legends for Data Sets S1 to S3. [file aem.01450-25-s0005.pdf]

Legends for Supplemental Data Sets

Antimicrobial-producing Bacteria from Fish Epidermal Mucus Alter the Fish Epidermal Bacterial Flora and Host Resistance to Infection

Hajime Nakatani, Naoto Suetake, and Katsutoshi Hori

Supplemental Data Set No. 1

- **Data Sheet 1. Amplicon sequence variants (ASVs) identified in the epidermal mucus and rearing water samples used for the isolation of *Pseudomonas mosselii* KH-ZF1.** This dataset summarizes the representative ASVs identified through 16S rDNA amplicon sequencing of three zebrafish epidermal mucus samples (zebrafish 1-3) and one rearing water sample, which served as microbial sources for the isolation of *Pseudomonas mosselii* KH-ZF1 and *Brevibacterium casei* C6. For each ASV, the table provides the representative nucleotide sequence, taxonomic classification based on a reference 16S rDNA database, and read counts for each sample.

- **Data Sheet 2. OTUs identified in the epidermal mucus following administration of strain KH-ZF1.** This dataset summarizes 16S rDNA amplicon sequencing results obtained from three or four epidermal mucus samples collected at day 0, day 1, day 2, day 4, and day7 after strain KH-ZF administration (KHd0-KHd7). For each OTU, the table provides the OTU identifier (name), taxonomic assignment based on a reference 16S rDNA database, combined abundance across samples, individual read counts per sample, and the representative reference sequence.

- **Data Sheet 3. OTUs identified in the gills and gut contents following the strain KH-ZF1 administration.** This dataset summarizes 16S rDNA amplicon sequencing results obtained from three or four gills and gut content samples collected at day 0, day 1 and day 3 after the strain KH-ZF administration (KHgilld0-KHgilld3 and KHgutd0-KHgutd3). For each OTU, the table provides

the OTU identifier (name), taxonomic assignment based on a reference 16S rDNA database, combined abundance across samples, individual read counts per sample, and the representative reference sequence.

- **Data Sheet 4. OTUs identified in the epidermal mucus during infection experiments.** This dataset summarizes 16S rDNA amplicon sequencing results obtained from epidermal mucus samples collected three days after pathogen challenge. Samples were obtained from eleven or twelve fish across the following treatment groups: pathogen challenge only (YrKHx0), pathogen challenge followed by a single dose of KH-ZF1 (YrKHx1), and pathogen challenge followed by a double dose of KH-ZF1 (YrKHx2). For each OTU, the table provides the OTU identifier (name), taxonomic assignment based on a reference 16S rDNA database, combined abundance across all samples, individual read counts per sample, and the representative reference sequence.

Supplemental Data Set No. 2

- **Data Sheet 5. OTUs identified in epidermal mucus from the control group of the strain KH-ZF1 administration experiment.** This dataset summarizes the 16S rDNA amplicon sequencing results from the control group, based on two or three epidermal mucus samples collected on days 1, 2, 3, and 6 of the strain KH-ZF1 administration experiment. For each OTU, the table provides the OTU identifier (name), taxonomic assignment according to a reference 16S rDNA database, combined abundance across samples, individual read counts per sample, and the representative reference sequence.

- **Data Sheet 6. OTUs identified in the epidermal mucus 24 hours after Fluviol C treatment.** This dataset summarizes 16S rDNA amplicon sequencing results obtained from four epidermal mucus samples collected 24 hours after treatment with Fluviol C at 12.5, 25, 50 ng /mL (24pi12.5, 24pi25, 24pi50) or untreated controls (24cont). For each OTU, the table provides the OTU identifier (name), taxonomic assignment based on a reference 16S rDNA database, combined abundance across samples, individual read counts per sample, and the representative reference sequence.

Supplemental Data Set No. 3

- **Data Sheet 7. OTUs identified in the epidermal mucus 48 hours after Fluviol C treatment.** This dataset summarizes 16S rDNA amplicon sequencing results obtained from four epidermal mucus samples collected 48 hours after treatment with Fluviol C at 50 ng /mL (48pi50) or untreated controls (48cont). For each OTU, the table provides the OTU identifier (name), taxonomic assignment based on a reference 16S rDNA database, combined abundance across samples, individual read counts per sample, and the representative reference sequence.

- **Data Sheet 8. Differential abundance analysis of OTUs in the epidermal mucus between Fluviol C-treated and untreated groups.** This dataset presents the results of differential abundance analysis of OTUs based on 16S rDNA amplicon sequencing data from Fluviol C-treated and untreated epidermal mucus samples at 24- and 48-hours post-treatment. For each OTU, the taxonomic assignment based on a reference 16S rDNA database (name), - Max group mean, $\log_2$ fold change, fold change, *p*-value, and FDR *p*-value.
